# TRBP and eIF6 Homologue in *Marsupenaeus japonicus* Play Crucial Roles in Antiviral Response

**DOI:** 10.1371/journal.pone.0030057

**Published:** 2012-01-18

**Authors:** Shuai Wang, An-Jing Chen, Li-Jie Shi, Xiao-Fan Zhao, Jin-Xing Wang

**Affiliations:** 1 The Key Laboratory of Plant Cell Engineering and Germplasm Innovation of Ministry of Education, School of Life Sciences, Shandong University, Jinan, Shandong, People's Republic of China; 2 Wuhan Institute of Virology, Chinese Academy of Science, Wuchang, Hubei, People's Republic of China; University of Hong Kong, Hong Kong

## Abstract

Plants and invertebrates can suppress viral infection through RNA silencing, mediated by RNA-induced silencing complex (RISC). Trans-activation response RNA-binding protein (TRBP), consisting of three double-stranded RNA-binding domains, is a component of the RISC. In our previous paper, a TRBP homologue in *Fenneropenaeus chinensis* (*Fc*-TRBP) was reported to directly bind to eukaryotic initiation factor 6 (*Fc*-eIF6). In this study, we further characterized the function of TRBP and the involvement of TRBP and eIF6 in antiviral RNA interference (RNAi) pathway of shrimp. The double-stranded RNA binding domains (dsRBDs) B and C of the TRBP from *Marsupenaeus japonicus* (*Mj*-TRBP) were found to mediate the interaction of TRBP and eIF6. Gel-shift assays revealed that the N-terminal of *Mj*-TRBP dsRBD strongly binds to double-stranded RNA (dsRNA) and that the homodimer of the TRBP mediated by the C-terminal dsRBD increases the affinity to dsRNA. RNAi against either *Mj*-TRBP or *Mj*-eIF6 impairs the dsRNA-induced sequence-specific RNAi pathway and facilitates the proliferation of white spot syndrome virus (WSSV). These results further proved the important roles of TRBP and eIF6 in the antiviral response of shrimp.

## Introduction

Viral diseases, especially white spot syndrome virus (WSSV) cause a great loss to the shrimp culture. Investigation of the antiviral mechanism of shrimp would help to control those diseases. Several studies demonstrate that the RNA interference (RNAi) play an important role in shrimp antiviral immunity [1].

Small interfering RNA (siRNA)/microRNA (miRNA)-induced RNA silencing has been demonstrated to be an important pathway regulating eukaryotic gene expression. This process depends on 20–25-nt siRNAs and miRNAs, guiding the RNA-induced silencing complex (RISC) to recognize and silence the target mRNA [2], [3], [4]. The core of RISC is composed of three proteins: Dicer, trans-activation response RNA-binding protein (TRBP), and Argonaute 2 (Ago2) [2], [3]. TRBP is a protein partner of the human Dicer and serve as a bridge between the double-stranded RNA (dsRNA) and Dicer, requiring for RNA silencing mediated by siRNAs/miRNAs [2], [3]. MacRae et al. used purified TRBP, Dicer, and Ago2 to assemble the RISC-loading complex *in vitro*. The complex shows dicing, slicing, guide-strand selection, and Ago2-loading activities [5], [6]. Introduction of those genes to *Saccharomyces cerevisiae* could reconstitute RNAi in budding yeast [7]. Eukaryotic initiation factor 6 (eIF6) is also a component of the human RISC, depletion of eIF6 abolish the miRNA-mediated silencing [8].

There is increasing evidence suggesting the crucial role of RNAi in invertebrate antiviral immunity. Significant amounts of dsRNA are produced following productive infection with positive-strand ssRNA, dsRNA, or DNA viruses [9]. These dsRNAs are thought to be subjected to the RNAi pathway, generating an antiviral response. The first direct evidence was reported in fruit fly, in which the flock house virus is both an initiator and a target of RNA silencing [10]. DsRNA-induced sequence-specific antiviral silencing and nonspecific immunity to white spot syndrome virus (WSSV), Taura syndrome virus, and yellow-head virus have also been reported in shrimp [1], [11], [12], [13].

TRBP belongs to the dsRNA-binding protein family, containing three dsRNA-binding domains (dsRBDs) [3], [14]. The first two dsRBDs bind to dsRNA with an affinity that is independent of dsRNA length and sequence [15], [16]. The second dsRBD of RDE-4, a TRBP homologue in *Caenorhabditis elegans*, plays a primary role in interaction with dsRNA [15]. The C-terminal dsRBD mediates binding to Dicer [3], [4], [17], protein kinase R (PKR) [18], and PKR-associated activator (PACT) [19], instead of binding to RNA. In our previous study, full-length cDNAs that encode a human TRBP2 homologue (*Fc*-TRBP) and an eIF6 protein (*Fc*-eIF6) in Chinese white shrimp(*Fenneropenaeus chinensis*) were cloned, and direct interaction between those two proteins was demonstrated. The proliferation of WSSV was reduced after injection of recombinant *Fc*-TRBP, implying a crucial role of *Fc*-TRBP in the antiviral defense response of shrimp [20]. However, the mechanism of antiviral silence in shimp remains obscure. In this study, different dsRNA-binding domains (dsRBDs) of TRBP from *Marsupenaeus japonicus* (*Mj*-TRBP) were expressed in *Escherichia coli*, and pull-down assays were performed to show which dsRBDs mediate the association with eIF6. Gel shift assays were performed to identify the dsRNA-binding activity of dsRBDs. Results would further characterize the dsRNA- and eIF6-binding activities of *Mj*-TRBP, and proving the important roles of *Mj*-TRBP and *Mj*-eIF6 in dsRNA-induced antiviral silencing.

## Materials and Methods

### Biological material

*M. japonicus* (about 10–15 g each) were bought from a shrimp market in Jinan, Shangdong Province, China and kept in tanks containing aerated seawater.

### Cloning of *Mj*-TRBP and *Mj*-eIF6 gene

Total RNA isolation and reverse transcription of the RNA were performed as described previously [20]. Two primers, MjTRBPF1 and MjTRBPR1, were designed according to the homologous gene sequences in order to obtain the complete open reading frame (ORF) of *Mj*-TRBP. The PCR procedure was as follows: 1 cycle at 94° C for 2 min, 35 cycles at 94° C for 30 s, 53° C for 45 s, and 72° C for 45 s and 1 cycle at 72° C for 10 min.

*Mj*-eIF6 was cloned using MjeIF6exF and MjeIF6exR primers (Table 1), which were designed based on homologous gene sequences. The PCR procedure was as follows: 1 cycle at 94° C for 2 min, 35 cycles at 94° C for 30 s, 53° C for 45 s, and 72° C for 45 s, and 1 cycle at 72° C for 10 min.

**Table 1:** Oligonucleotide primers used in this study.

Primer Name	Nucleotide Sequence (5′- 3′)
Gene cloning	
MjTRBPF1	TCATGTATCATCAACCTCCACCAAA
MjTRBPR1	CCCCTTCTCCCTAACACAAAGT
Recombinant expression	
MjeIF6exF	TACTCA GAATTC(*Eco*R I)ATGGCTGTTCGCTGTCAGTTT
MjeIF6exR	TACTCACTCGAG(*Xho* I)GAAGGCTGGTTTCCCTCACTTTA
MjTRBPexF1	TACTCAGAATTC(*Eco*R I)ATGTATCATCAACCTCCACCAA
MjTRBPexR1	TACTCACTCGAG(*Xho* I)TTAATCGTATGGGGAAACAATCTG
MjTRBPexF2	TACTCAGAATTC(*EcoR* I)ACCCAGATTGTTTCCCCATAC
MjTRBPexR2	TACTCACTCGAG(*Xho* I)TTAATCGTCGTCATCAGCCAAGA
MjTRBPexF3	TACTCAGAATTC(*Eco*R I)TCGCTGAGCAACGTGAACCT
MjTRBPexR3	TACTCACTCGAG(*Xho* I)CCCCGGCACAAACTTTACTT
DsRNA preparation	
MjTRBPiF	GCGTAATACGACTCACTATAGG(T7)CAGATAGAGGGCGCAGTTCA
MjTRBPiR	GCGTAATACGACTCACTATAGG(T7)CATCGTCGTCATCAGCCAAG
MjeIF6iF	GCGTAATACGACTCACTATAGG(T7)ATGGCTGTTCGCTGTCAGTTT
MjeIF6iR	GCGTAATACGACTCACTATAGG(T7)GGCTGGTTTCCCTCACTTTA
GFPiF	GCGTAATACGACTCACTATAGG(T7)TGGTCCCAATTCTCGTGGAAC
GFPiR	GCGTAATACGACTCACTATAGG(T7)CTTGAAGTTGACCTTGATGCC
VP28F	GCGTAATACGACTCACTATAGG(T7)CACTCTTTCGGTCGTGTCGG
VP28R	GCGTAATACGACTCACTATAGG(T7)TCACAGGAATGCGGAGGTTT
MjPOiF	GCGTAATACGACTCACTATAGG(T7)AGCGGTCAGCGAGGAATAGA
MjPOiR	GCGTAATACGACTCACTATAGG(T7)GGTGAGCATGAAGAAAAGTTGGA
Real time PCR	
MjTRBPiF3	GGTATCCCAGGGAACCCAGTT
MjTRBPiR2	CATCGTCGTCATCAGCCAAG
MjeIF6RTF	AACTCCATTGCTGCTGGTCTG
MjeIF6RTR	TACTGTCGATGAGTGCGTTTCTC
MjPORTF	GGATCTGCCTTCTCCTTCTTCC
MjPORTR	TAGCATCCAGGAGTCGAGATCG
ActinF	AGTAGCCGCCCTGGTTGTAGAC
ActinR	TTCTCCATGTCGTCCCAGT

*Eco*RI, *Xho* I sites, and T7 promoters are underlined in the forward and reverse primers.

### Recombinant expression of different dsRBDs of *Mj*-TRBP

The three dsRBDs of *Mj*-TRBP were denoted as TRBP-DA, TRBP-DB, and TRBP-DC. TRBP-DA (369 bp), TRBP-DB (294 bp), TRBP-DC (257 bp), TRBP-DAB (639 bp, containing TRBP-DA and TRBP-DB), and TRBP-DBC (701 bp, containing TRBP-DB and TRBP-DC) fragments of *Mj*-TRBP were amplified from hemocyte cDNA using the primers: MjTRBPexF1 and MjTRBPexR1, MjTRBPexF2 and MjTRBPexR2, MjTRBPexF3 and MjTRBPexR3, MjTRBPexF1 and MjTRBPexR2, MjTRBPexF2 and MjTRBPexR3, and MjeIF6exF and MjeIF6exR, respectively (Table 1). *Eco*RI and *Xho*I restriction sites were inserted at the beginning and end of the DNA fragments, so that the PCR products could be cloned into the *Eco*RI and *Xho*I restriction sites of pET-30a. Recombinant plasmids were transformed into *E. coli* BL21 (DE3) cells, which were then cultured in Luria-Bertani medium with 25 µg/ml ampicillin. When the OD_600_ of the culture reached 0.5, isopropyl-β-D-thiogalactoside (IPTG; 0.1 mM) was added. After 3 h of culture, cells were collected by centrifugation at 6000 rpm for 10 min. They were then resuspended in PBS (140 mM NaCl, 2.7 mM KCl, 10 mM Na_2_HPO_4_, and 1.8 mM KH_2_PO_4_) containing 0.2% Triton X-100. Following cell sonication and centrifugation, proteins were purified using His-Bind resin (Novagen), according to the manufacturer's instructions. The purified proteins were analyzed by 15% SDS-PAGE and stained by Coomassie brilliant blue G250.

### Pull-down assays

The pull-down assay was performed as described previously [20]. Briefly, TRBP-DA with an N-terminal His tag was expressed in *E. coli*. After induction by IPTG and sonication, 10 ml lysed cells were incubated with 1 ml His-Bind Resin for 5 min and washed with 10 ml binding buffer (0.5 M NaCl, 20 mM Tris-Cl (pH 7.9), 5 mM imidazole) and then 6 ml washing buffer (0.5 M NaCl, 20 mM Tris-Cl (pH 7.9), 30 mM imidazole). About 200 µg purified eIF6 with the N-terminal His tag removed by incubation with thrombin (eIF6ΔHis-tag) was added and incubated with the His-tagged protein and His-Bind Resin for 10 min at 4° C. EIF6ΔHis-tag and eIF6 with His-tag were analyzed by SDS-PAGE, and the difference in mobility was observed, ensuring the His-tag of eIF6ΔHis-tag was completely removed. After 3 washes with 10 ml washing buffer, proteins were eluted with eluting buffer (0.5 M NaCl, 20 mM Tris-Cl (pH 7.9), 1 M imidazole). The eluted proteins were analyzed by 15% SDS-PAGE. Pull-down assays were also performed to identify the interaction between TRBP-DB and eIF6, TRBP-DC and eIF6, TRBP-DAB and eIF6, TRBP-DBC and eIF6, TRBP-DC and TRBP, TRBP-DC and TRBP-DAB, and TRBP-DAB and TRBP.

### dsRNA preparation

dsRNA was prepared as described previously [21], with slight modifications. DNA fragments were amplified using MjTRBPiF and MjTRBPiR (543 bp), MjeIF6iF and MjeIF6iR (802 bp), MjPOiF and MjPOiR (810 bp),GFPiF and GFPiR (467 bp) primers, respectively (Table 1). A T7 promoter was linked to both ends of the PCR products. After extraction with phenol/chloroform and precipitation with ethanol, DNA fragments were used as templates for dsRNA synthesis.

Transcription was carried out as follows: 8 µg DNA templates was mixed with 25 µL 5× transcription buffer, containing 80 U T7 RNA polymerase (Fermentas, USA), 3 µL 10 mM A/U/C/GTP each (Fermentas, USA), and 120 U RNasin (TaKaRa, Japan). RNase-free water was added to a volume of 125 µL. After incubation at 37° C for 4 h, dsRNAs were incubated at 75° C for 5 min and then cooled to room temperature for annealing. To remove the template, 40 U RNase-free DNase I (Fermentas, USA) was added and the solution was incubated at 37° C for 30 min. After extraction with phenol/chloroform and precipitation with ethanol, dsRNAs were resuspended in 60 µL RNase-free water. The dsRNA purity and integrity were determined using agarose gel electrophoresis. The dsRNAs were quantified using a spectrophotometer (GeneQuant; Amersham Biosciences).

### Gel-shift assay

*Mj*-eIF6 dsRNA was prepared as above, with additional 0.35 µL 10 mM digoxigenin (Dig)-labeled UTP (Roche Applied Sciences) added to the transcription buffer during preparation. The VP28 dsRNA was also prepared using the DNA template amplified with VP28F1 and VP28R1 (193 bp) primers from the genomic DNA of WSSV. All components of the reaction were added to 20 µL PBS, including: 10 mM DTT, 10 U RNasin (TaKaRa, Japan), 2.5 µg Dig-labeled *Mj*-eIF6 dsRNA (802 bp) or 0.5 µg Dig-labeled VP28 dsRNA (193 bp), and 0.5 µg protein (dsRBDs). The mixture was incubated at 4° C for 30 min to allow binding and was subsequently loaded onto to a 5% native polyacrylamide gel. After electrophoresis, proteins and Dig-labeled dsRNA were transferred to a nylon membrane, and an anti-Dig-phosphatase antibody (AB) was used to detect dsRNA. AB binding was visualized by incubation of the membrane with 5-bromo-4-chloro-3-indolyl phosphate and nitro blue tetrazolium chloride. Recombinant adenylate kinase 1 (expressed in the same *E. coli* system, a gift of from Dr. Weiwei Zheng, paper is under preparation) from *Helicoverpa armigera* (*Ha*-AK) was used as a control.

### In vivo RNAi assay

RNAi assay was performed according to a previously described method [21]. Briefly, 60 µg of *Mj*-TRBP dsRNA, *Mj*-eIF6 dsRNA, *Mj*-prophenoloxidase (*Mj*-PO) dsRNA or control dsRNA GFP was injected into the abdominal segment of *M. japonicus*. Injection was repeated 24 h after the first injection. The total RNA from hemocytes was extracted at 24 h post the second injection. Transcription of *Mj*-TRBP *Mj*-eIF6 and *Mj*-PO were detected by quantitative real time PCR to confirm knockdown using the MjTRBPiF3 and MjTRBPiR2, MjeIF6RTF and MjeIF6RTR, MjPORTF and MjPORTR primers (Table 1).

To investigate the role of *Mj*-TRBP and *Mj*-eIF6 in the RNAi pathway, shrimps were injected with dsRNA against *Mj*-TRBP, *Mj*-eIF6 and GFP(control) as above. To examine whether the activity of RNAi pathway was impaired by the silencing of these protein, *Mj*-PO dsRNA was subsequently injected to the shrimp. Transcription of *Mj*-PO was analyzed by quantitative real time PCR.

### Functional analysis of *Mj*-TRBP and eIF6

The preparation and quantification of WSSV inocula were done as described previously [20]. *M. japonicus* subjects were divided into 3 groups and injected with 60 µg *Mj*-TRBP dsRNAs, *Mj*-eIF6 dsRNAs, and *Mj*-GFP dsRNAs, as above. A second injection was done 24 h later. WSSV (1.6×10^8^ copies/shrimp) were injected into the abdominal segment of shrimps 24 h after the second injection of dsRNA. At 36 h post-WSSV-infection, genomic DNA was extracted from the gills of the shrimp, and quantitative RT-PCR was performed to quantify WSSV.

In another experiment, one group of shrimp were injected with WSSV (8.0×10^7^ copies/shrimp) and 8 µg of *Mj*-TRBP, while the other groups received the same amounts of WSSV and 8 µg of TRBP fragments (DA, DB, DC, DAB and DBC). The control group was injected with WSSV and 8 µg of *Ha*-AK2. At 24 h post injection, genomic DNA was extracted from shrimp gills, and quantitative real time PCR was performed to quantify WSSV.

## Results

### Cloning of TRBP and eIF6 in *M. japonicus*

*Mj*-TRBP was cloned from *M. japonicus*, and the complete ORF is composed of 1032 bp encoding a 343-aa protein. The cDNA sequence of *Mj*-TRBP was deposited in GenBank (accession no. HM149250). Alignment revealed high sequence similiarity between *Mj*-TRBP and the homologue in *F. chinensis* (99.42% identity) (Fig. S1). Two additional isoforms of *Mj*-TRBP, *Mj*-TRBP2 (GenBank accession no. HM149251) and *Mj*-TRBP 3 (GenBank no. HM149252), were also identified. These isoforms are identical except for the lack of 60 and 141 bp between the second and the third dsRBDs.

The homologue of eIF6 in *M. japonicus* was cloned (GenBank no. HM149253). The complete ORF of *Mj*-eIF6 is composed of 738 bp encoding a 245-aa protein. Alignment analysis showed that *Mj*-eIF6 is highly homologous to *Fc*-eIF6 (97.55% identity, Fig. S2).

### TRBP-DB and TRBP-DC mediate the interaction between TRBP and eIF6 in shrimp

**Figure 1:**
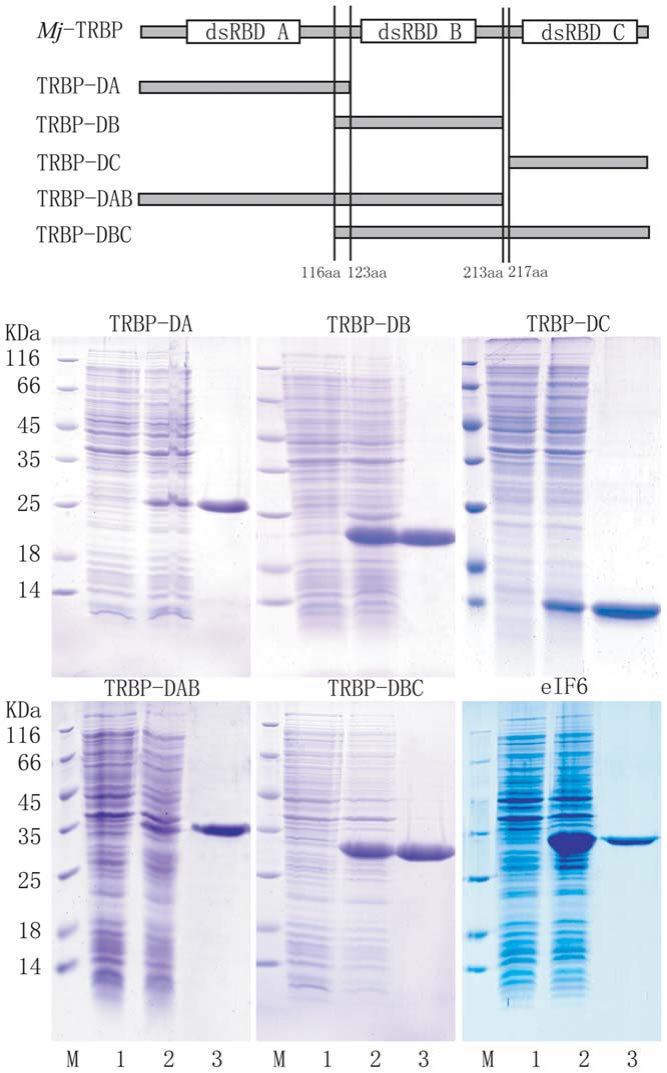
Scheme of the dsRBDs of *Mj*-TRBP and the SDS-PAGE analysis of recombinant dsRBDs of *M*j-TRBP and *Fc*-eIF6 expressed in *E. coli*. The N-terminal (first), middle (second), and C-terminal (third) dsRBDs were denoted as dsRBD A, dsRBD B, and dsRBD C, respectively. The recombinant protein TRBP-DA, TRBP-DB, TRBP-DC, TRBP-DAB, and TRBP-DBC contain dsRBD A, dsRBD B, dsRBD C, dsRBD A and dsRBD B, and dsRBD B and dsRBD C,respectively. Lane M, molecular mass marker; lane 1, total protein obtained from *E. coli*; lane 2, total protein obtained from expressing strains induced by IPTG; lane 3, recombinant proteins purified by His-Bind resin chromatography.

**Figure 2:**
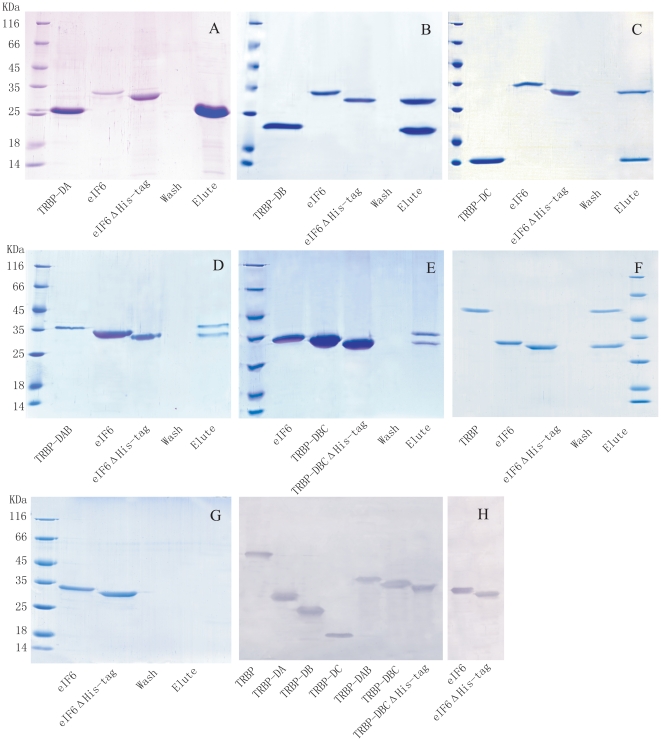
Pull-down assays that identify interactions between dsRBDs of *Mj*-TRBP and *Fc*-eIF6. A: Recombinant His-tagged TRBP-DA was incubated with His-Bind resin, to which eIF6ΔHis-tag (the His tag was removed from recombinant eIF6 by thrombin treatment and SDS-PAEG show the different mobility of eIF6 and eIF6ΔHis-tag, ensuring the His-tag of eIF6ΔHis-tag was removed completely) was added and incubated for 10 min. Wash buffer did not elute either protein, and elution buffer only eluted TRBP-DA, indicating the incapability of TRBP-DA to bind eIF6. B, C, D, E and F: The interactions between eIF6 and TRBP-DB, eIF6 and TRBP-DC, eIF6 and TRBP-DAB, eIF6 and TRBP-DBC eIF6 and full length TRBP (positive control) were identified by Pull down assays, respectively. G: The eIF6ΔHis-tag was incubated with His-Bind resin, and no protein was eluted after wash. H: To verify those proteins, western blot analysis was performed using TRBP (left) or eIF6 (right) specific antibody. The results indicated that eIF6 could be co-eluted with those TRBP fragments containing the second and third dsRBD, which indicated that the TRBP-DB and -DC, but not TRBP-DA mediated the interaction between TRBP and eIF6.

We previously reported the interaction between TRBP and eIF6 in shrimp by screening the T7 phage display library and confirming by pull-down assay [20]. Similar to other members of the TRBP family, *Mj*-TRBP contains three dsRBDs, denoted as TRBP-DA (27–92 aa), TRBP-DB (130–196 aa) and TRBP-DC (272–338 aa), and it remains unclear which domains mediate the interaction with eIF6. To address this, different domains of *Mj*-TRBP were recombinant-expressed and purified (Fig. 1). His-tag pull-down assays were performed to identify the interaction between these dsRBDs and *Fc*-eIF6. In Fig. 2A, His-tagged TRBP-DA was incubated with His bind resin, and subsequently the resin was incubated with eIF6ΔHis-tag (the His tag of eIF6 was removed by treatment with thrombin *in vitro*). SDS-PAEG show the different mobility of eIF6 and eIF6ΔHis-tag, and ensure the His-tag of eIF6ΔHis-tag was removed completely. After stringent wash, only TRBP-DA was eluted, indicating TRBP-DA could not bind eIF6. While eIF6ΔHis-tag was proved to be unable to bind His bind resin non-specifically (Fig. 2G). Pull-down assays were also performed between eIF6 and TRBP-DB/DC/DAB/DBC, and both eIF6 and TRBP-DB/DC/DAB/DBC could be eluted (Fig. 2B,C,D,E). Full length TRBP was used as positive control (Fig. 2F). All the proteins were verified by *Fc*-TRBP or *Fc*-eIF6 antibody (Fig. 2H). Results indicated that all dsRBDs could bind to eIF6, except TRBP-DA. Therefore, both TRBP-DB and TRBP-DC mediate the association of TRBP with eIF6 (Fig. 2).

### TRBP-DA has a higher affinity with dsRNA, and the TRBP-DC can enhance the dsRNA-binding activity of TRBP

**Figure 3:**
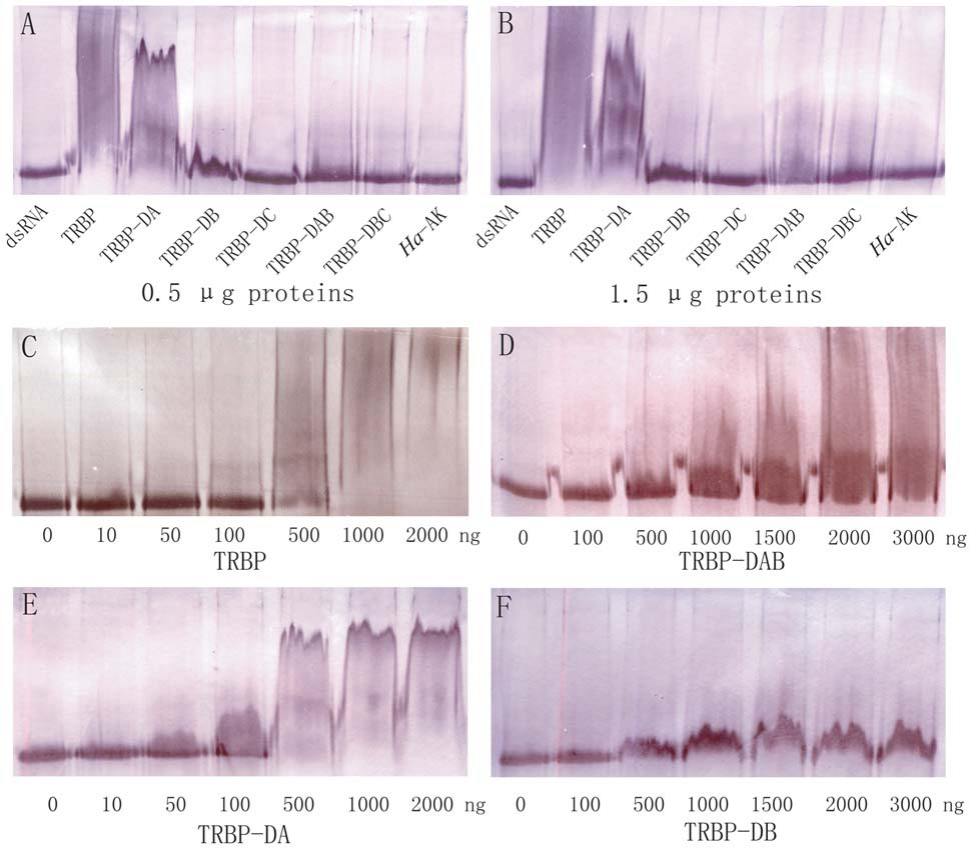
Gel-shift assays that identify the dsRNA-binding activity of recombinant dsRBDs of *Mj*-TRBP and full length recombinant *Fc*-TRBP. A, B: 0.5 µg (A) and 1.5 µg (B) recombinant protein were incubated with 2.5 µg dsRNA (802 bp). *Ha*-AK served as control. C, D, E, F: Increasing amounts of recombinant protein (TRBP, TRBP-DAB, TRBP-DA, and TRBP-DB) were added and incubated with 2.5 µg dsRNA; the number under each lane represents the amount of recombinant proteins added to the dsRNA. *Fc*-TRBP and dsRBD A of *Mj*-TRBP could bind to dsRNA with high affinity. The affinity of dsRBD B was very weak. dsRBD C was unable to bind to dsRNA.

**Figure 4:**
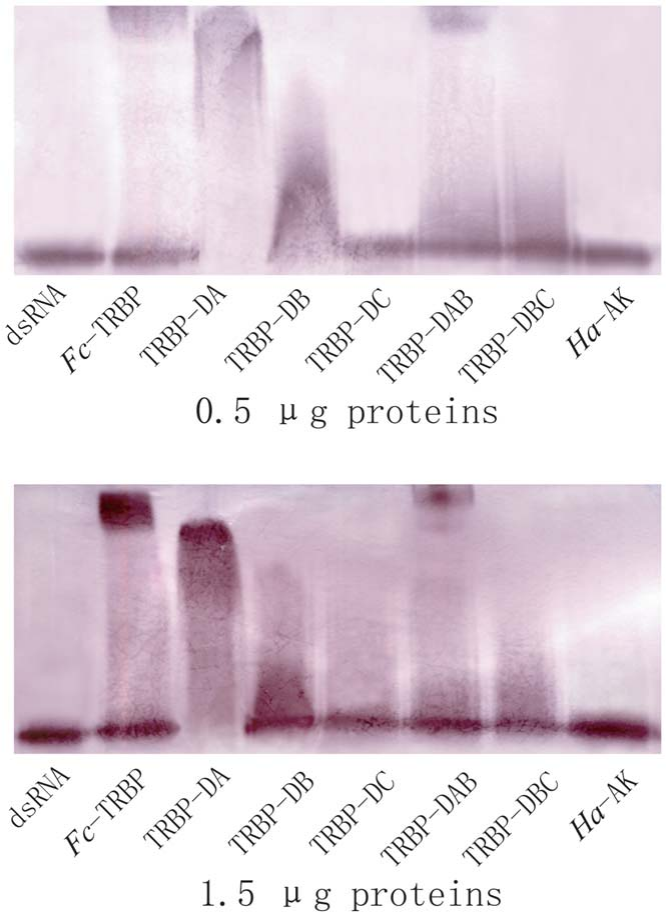
Gel-shift assays that identify the dsRNA-binding activity of recombinant dsRBDs of *Mj*-TRBP and full length recombinant TRBP 0.5 µg (up) and 1.5 µg (down) protein were incubated with 2.5 µg 193-bp dsRNA. *Ha*-AK served as the control.

To investigate the dsRNA binding activity of *Mj*-TRBP, recombinant, gel-shift experiments were performed using 0.5–1.5 µg proteins (TRBP, dsRBDs or *Ha*-AK as a control) and 2.5 µg Dig-labeled *Mj*-eIF6 dsRNA (802 bp). The results show that TRBP and TRBP-DA have a high affinity for dsRNA, and TRBP-DB and BC only weakly bind to dsRNA. TRBP-DAB has a medium affinity between TRBP-DA and B. TRBP-DC is completely unable to bind to dsRNA (Fig. 3 A, B). Different concentrations of TRBP, TRBP-DAB, TRBP-DA, and TRBP-DB were subjected to the binding affinity assay, and the results revealed that the binding activity of those proteins to dsRNA occurs in a dose dependent manner (Fig. 3C, D, E, F). Gel-shift assays were repeated using 193 bp Dig-labeled VP28 dsRNA (from WSSV), and the results confirmed that TRBP-DA has a high affinity for dsRNA, TRBP-DB has a weak affinity, and TRBP-DC cannot bind to dsRNA (Fig. 4). These results also suggest that the TRBP-binding activity is independent of dsRNA sequence.

**Figure 5:**
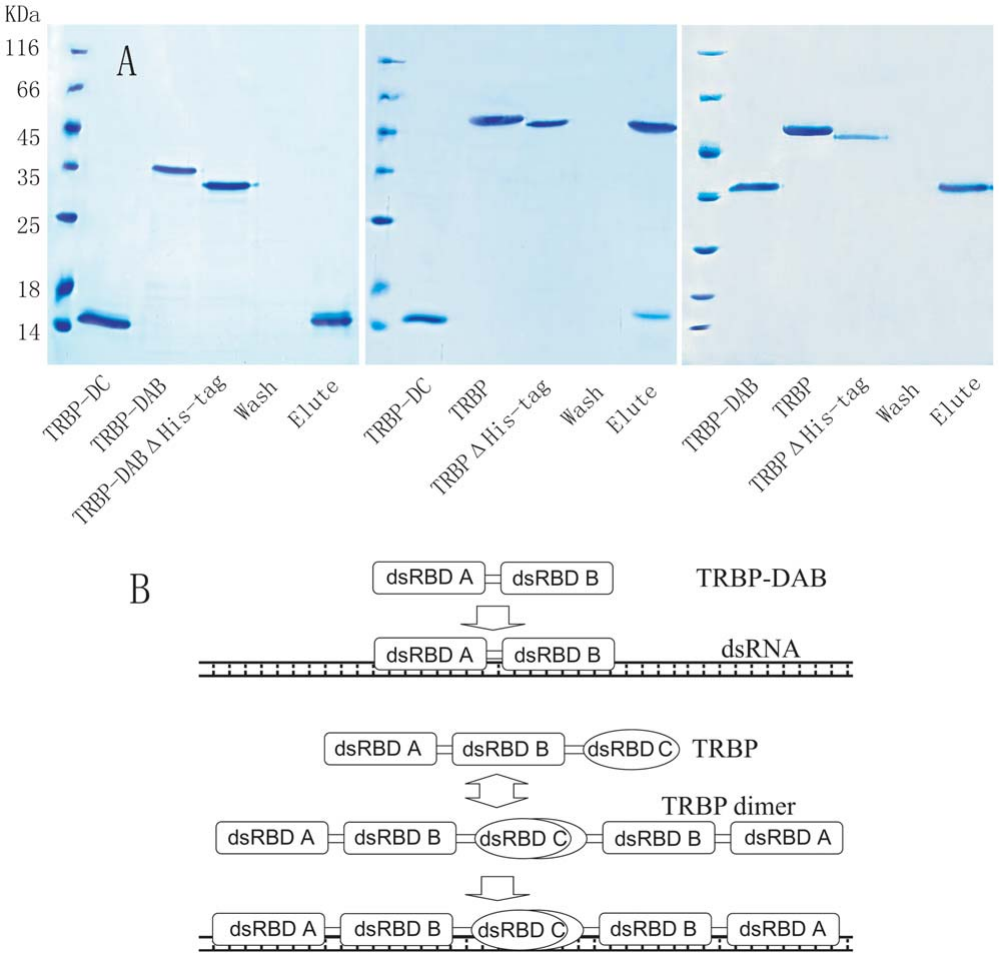
A: Pull-down assays showing that TRBP-DC mediates the homo-dimerization of TRBP in shrimp. Left: Recombinant His-tagged TRBP-DC was incubated with His-Bind resin, and then TRBP-DABΔHis-tag was added. After stringent wash, only TRBP-DC was eluted, indicating TRBP-DAB could not bind to TRBP-DC. Middle: Pull down assay was performed to identify the interaction between TRBP-DC and full length TRBP, and both proteins could be eluted, suggesting the interaction between TRBP-DC and full length TRBP. Right: pull down assay show TRBP-DAB could not bind to full length TRBP. These results indicate that TRBP-DC mediates the dimerization of full length TRBP. B: Illustration of the differences in affinity of full length TRBP and TRBP-DAB to dsRNA in shrimp. TRBP-DAB binds dsRNA uncooperatively and exhibits lower affinity. C-terminal dsRBD of TRBP mediate the dimerization of TRBP, resulting in a higher affinity of TRBP to dsRNA compared to TRBP-DAB.

Both full length TRBP and TRBP-DAB contain dsRBD A and dsRBD B, so they were expected to have equal affinity for dsRNA, because of the incapability of dsRBD C to bind dsRNA. However, results showed that the dsRNA affinity of full length TRBP is much higher than that of TRBP-DAB (Fig. 3A, B, C, D). One possible explanation is that the C-terminal region of TRBP assists in the association of other dsRBDs with dsRNA, although it is incapable of binding to dsRNA by itself. Pull-down assays showed that TRBP-DC can interact with full length TRBP and that there is no association observed between TRBP-DC and TRBP-DAB and between full length TRBP and TRBP-DAB (Fig. 5A).

Therefore, TRBP-DC seems to mediate the dimerization of TRBP. As a result, TRBP could form dimmer via the C-terminal dsRBD and bind to dsRNA co-operatively, exhibiting a significantly higher affinity to dsRNA than TRBP-DAB, which was lack of the C-terminal region and could not form dimer (Fig. 5B). Native PAGE was performed to confirm the dimerization of TRBP-DC (Fig. S3). The dimer of TRBP-DC exhibits smear bands with a decreased migration compared to TRBP-DC monomer. The result might suggest that the TRBP-DC could form different polymers in vitro.

### TRBP and eIF6 are involved in dsRNA-induced RNA silencing

**Figure 6:**
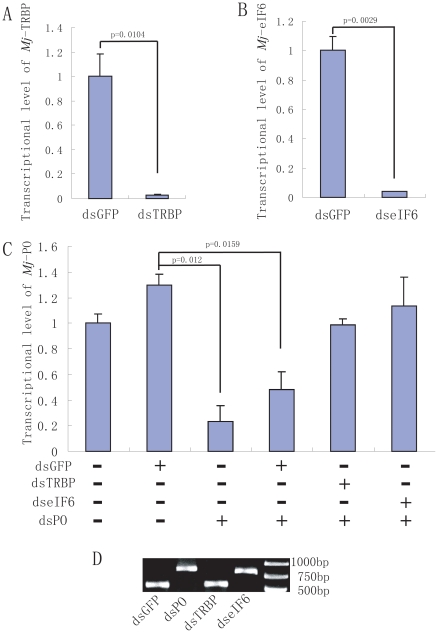
*M*j-TRBP and *Mj*-eIF6 were involved in dsRNA-induced silencing. A, B; Injection of *Mj*-TRBP dsRNA (dsTRBP) or *Mj*-eIF6 dsRNA (dseIF6) could significantly knock down *Mj*-TRBP or *Mj*-eIF6, respectively. C: Silencing of *Mj*-TRBP and *Mj*-eIF6 impaired the activity of the RNAi pathway. Injection of *Mj*-PO dsRNA (dsPO) could silence *Mj*-PO, whereas, the mRNA level of *Mj*-PO still remained high when the shrimp were injected with dsTRBP and dseIF6, but not dsGFP, indicating the activity of the RNAi pathway was impaired by knocking down *Mj*-TRBP or *Mj*-eIF6. GFP dsRNA was used as a control. D: Agarose gel electrophoresis show the same amount of dsRNA was injected into the shrimp.

In our previous study, TRBP was show to be involved in antiviral innate immunity of shrimp [20]. Given the fact that both TRBP and eIF6 is components of RISC, we further investigated the roles of TRBP and eIF6 in dsRNA-induced silencing in shrimp. To clarify this question, dsRNA was prepared to knockdown the *Mj*-TRBP or *Mj*-eIF6. In Fig. 6 A and B, injection of *Mj*-TRBP or *Mj*-eIF6 dsRNA (dsTRBP, dseIF6) can significantly knock down the transcription of *Mj*-TRBP or *Mj*-eIF6 in shrimp respectively, comparing to the shrimp injected with GFP dsRNA (dsGFP).

To examine whether silencing of *Mj*-TRBP, *Mj*-eIF6 affects the RNAi pathway, the dsRNA of *Mj*-prophenoloxidase (dsPO) was prepared, and then injected with or without dsTRBP, dseIF6 and dsGFP. In Fig. 6D, agarose gel electrophoresis were performed to ensure the same amount of dsRNA was injected. Injection of dsPO could significantly down regulate mRNA level of *Mj*-PO. However, the mRNA level of *Mj*-PO still remained high when the dsPO were injected with either dsTRBP or dseIF6 (Fig. 6 C), implying dsRNA induced RNA silence was impaired by knocking down TRBP or eIF6. Therefore, our results indicated that both *Mj*-TRBP and *Mj*-eIF6 played crucial roles in the dsRNA/siRNA induced RNA silence.

### TRBP and eIF6 are involved in dsRNA-induced antiviral silencing

**Figure 7:**
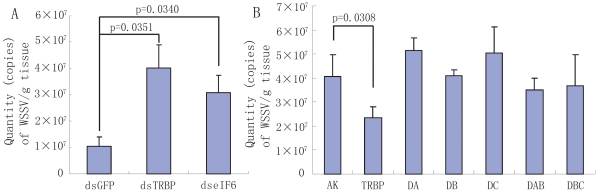
*Mj*-TRBP and *Mj*-eIF6 involvement in antiviral immunity. A: The proliferation of WSSV was enhanced by silencing *Mj*-TRBP and *Mj*-eIF6. B: Injection of recombinant TRBP inhibited the proliferation of WSSV, whereas injection of TRBP fragments did not, suggesting the integrity of TRBP is required for its antiviral function.

To further study the roles of TRBP and eIF6 in antiviral silencing, WSSV was injected into shrimp that had been previously injected with *Mj*-TRBP, *Mj*-eIF6, or GFP dsRNA. Results showed that the DNA copies of WSSV in both the TRBP-silenced group (about 4.03×10^7^ copies/g tissues) and the eIF6-silenced group (about 3.08×10^7^ copies/g tissues) were greatly increased compared to the group injected with GFP dsRNA (about 1.04×10^7^ copies/g tissues) at 36 h post injection (Fig. 7A), suggesting the important roles of *Mj*-TRBP and *Mj*-eIF6 in antiviral silencing of shrimp.

In another experiment, recombinant proteins (*Fc*-TRBP, TRBP-DA, DB, DC, DAB, DBC, *Ha*-AK as control) and WSSV (8.0×10^7^ copies/shrimp) were co-injected into the shrimp. After 24 h, the titer of WSSV decreases remarkably in shrimp injected with TRBP (about 2.33×10^7^ copies/g tissues) compared with the control group (about 4.06×10^7^ copies/g tissues) (Fig. 7B), whereas the replication of WSSV in shrimp injected with TRBP fragments is not significantly different from the control group, implying that the full length of TRBP is required for the antiviral function.

## Discussion

In the RNAi pathway, dsRNA was processed into siRNA by Dicer, and then siRNA was assembled into large ribonucleoprotein complexes called RISCs, the core of which is composed of Dicer, Ago2, and TRBP [2], [22], [23]. The siRNA-induced gene silencing mechanism is conserved in a wide range of eukaryotic organisms from plants to mammals. Since the discovery of dsRNA-mediated RNAi in *C. elegans* in 1998 [24], more evidence have supported the important roles of RNAi in invertebrates, plants, and fungi [25]. DsRNAs produced by viruses during their infectious cycles [9] serve as the substrate of Dicer, thus triggering antiviral RNAi response. Blocking RNAi pathway leads to higher virus susceptibility and lethality in *Drosophila* [26], [27], [28]. DsRNA-mediated antiviral RNAi has been reported in plants, flies, worms, and shrimps [1], [11], [25]. In this study, TRBP and eIF6 were proven to be involved in the dsRNA-induced RNAi pathway. Silence of either TRBP or eIF6 significantly impairs the RNAi pathway and increases WSSV replication. These results are similar to reports in *Drosophila* [10]. A previous paper has proven the role of eIF6 in miRNA-mediated post-transcriptional silencing in humans [8]. Results of the present study indicate that *Mj*-eIF6 also plays a crucial role in RNAi pathway.

TRBP stabilize the Ago2–Dicer interaction, and Dicer, TRBP, and Ago2 are required for siRNA- and miRNA-mediated RNAi [2], [4], [6], [8], [29], [30]. Chendrimada et al. isolated a large TRBP-containing complex that includes Dicer, Ago2, MOV10, eIF6, and 60S ribosome proteins [8], [31], [32]. The C-terminal domain of human TRBP (referred to as the Medipal domain) mediates protein-protein interactions with PKR, Dicer, PACT etc [19], [33], [34], [35], [36], [37]. Results of this study showed that TRBP-DB and TRBP-DC mediate the association of *Mj*-TRBP with eIF6 in shrimp. The dsRBD C of *Mj*-TRBP is very similar to the Medipal domain of human TRBP and is suggested to have a similar function in protein binding (Fig. S4). The C-terminal of TBBP is reported to contain a 69aa Dicer binding site [17], mediating the association with Dicer, and is essential for antiviral RNAi. Our results are consistent with Parker's report [38], in which the C-terminal of RDE-4 is required for Dicer activity in *C.elegans*, and the mutant in C-terminal of RDE-4 block siRNA production. Our results show that injection of the TRBP protein inhibits the proliferation of virus, but injection of TRBP-DAB not, implying a role of C-terminal dsRBD of TRBP in antiviral immunity. Our results also indicate that the full length of TRBP is required for the antiviral function of TRBP.

The second dsRBD of human TRBP contains a KR-helix motif that can strongly bind to dsRNA, whereas N-terminal dsRBD minimally binds RNA and directs guide strand selection from microRNA duplexes in humans [35], [39]. Yamashita et al. found that the structure of dsRBD1 and dsRBD2 of human TRBP were similar, and could bind to siRNA, with dissociation constants of 220 and 113 nM respectively [40]. A lysine- and arginine-rich motif termed TR13 (KKLAKRNAAAKMLLR) derived from the dsRBD2 of human TRBP is necessary to bind HIV trans-activation response(TAR) RNA upper-stem/loop site, and the two Arg and Lys residues are important for the RNA-binding activity [35], [36], [37]. However, results of this study showed that the N-terminal dsRBD of *Mj*-TRBP has a high dsRNA affinity and TRBP-DB can only weakly bind to dsRNA. There are 5 different residues in the equivalent peptides of TRBP-DB (KKLAKRQAAYKMTQL) and TR13, where the replacement of an Arg (R) by Leu (L) might lead to the low dsRNA affinity of TRBP-DB (Fig. S3 boxed sequence). The equivalent peptide in TRBP-DA (KKKAKHAAAKAVL) does not contain Arg. Further study is needed to clarify the high dsRNA affinity of TRBP-DA.

The gel-shift assays showed that the dsRNA affinity of full length TRBP is much higher than the affinity of TRBP-DAB, and then pull-down assays showed that TRBP can form dimers in the C-terminal region (TRBP-DC), allowing for the cooperative binding of *Mj*-TRBP to dsRNA (Fig. 5B). TRBP is capable of binding to itself to form dimer [3], [41], [42]. Paker et al. reported that RDE-4, a TRBP homologue in *C. elegans*, is a homodimer in solution, and the C-terminal domain of RDE-4 is required for dimerization. RDE-4 displays higher affinity to longer dsRNA because multiple RDE-4 form long clusters along the dsRNA [15], [38]. Results of this study showed that TRBP in shrimp is homo-dimerized by the C-terminal region and display a higher affinity to dsRNA than TRBP-DAB.

In conclusion, this study found that the TRBP-DA of *Mj*-TRBP strongly binds to dsRNA, while TRBP-DB only weakly binds to dsRNA. The C-terminal region (TRBP-DC) increases TRBP affinity to dsRNA by mediating dimerization of TRBP and that TRBP-DB and TRBP-DC mediate interaction with eIF6. Both *Mj*-TRBP and *Mj*-eIF6 are necessary for dsRNA-induced silencing and play important roles in antiviral immunity in shrimp.

## Supporting Information

Figure S1 Multiple alignments of *Fc*-TRBP1 (GenBank no. EU679001) with *Mj*-TRBP1-3.
(JPG)

Figure S2 Multiple alignments of *Fc*-eIF6 (GenBank no. EU679001) with *Mj*- eIF6.
(JPG)

Figure S3 Native PAGE and Western blot was performed to confirm the dimerization of TRBP-DC(left panel). After Native PAGE of TRBP-DA,-DB and DC, the proteins in the PAGE gel were transferred onto a nitrocellulose membrane and were detected with TRBP antibody. TRBP-DA amd DB were used as control. TRBP-DC dimer exhibits a smear bands above the band of DC monomer. SDS-PAGE and Western blot were performed to verify the identity of the bands (right panel).
(JPG)

Figure S4 Multiple alignments of *Mj*-TRBP (GenBank no. HM149251) amino acid sequences from other animals. The TR13 sequence of human TRBP and the equivalent peptides in other animal are marked with a box. The following sequences were selected from GenBank: *F. chinensis* (EU679001), *Aedes aegypti* (XP_001659426.1), *Danio rerio* (NP_956291.1), *Drosophila melanogaster* (NP_609646.1), *Homo sapiens* (AAP36873.1), *Mus musculus* (AAH02028.1), *Rattus norvegicus* (NP_001030113.1), *Xenopus tropicalis* (NP_001025646.1). Dark shadow: identity = 100%; Grey shadow: identity≥75%; Light grey shadow: identity≥50%. The alignment was performed by DNAman 3.1.
(JPG)
