# Supplementary figures and images for: TRBP and eIF6 Homologue in Marsupenaeus japonicus Play Crucial Roles in Antiviral Response
Source: PLoS One. 2012 Jan 18;7(1):e30057. doi: 10.1371/journal.pone.0030057 (PMC3261181; doi:10.1371/journal.pone.0030057)

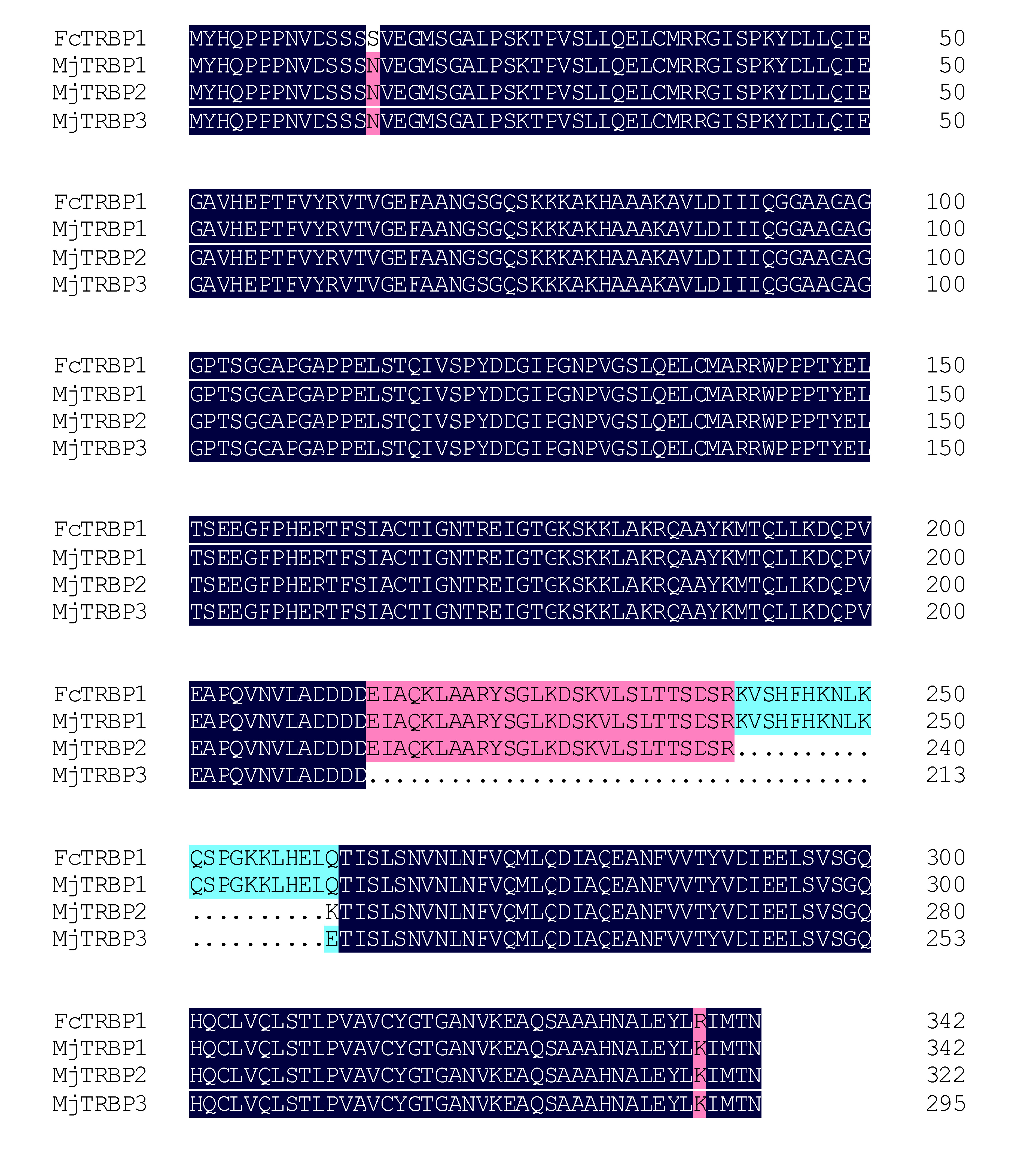

Supplement: Figure S1 — Multiple alignments of Fc-TRBP1 (GenBank no. EU679001) with Mj-TRBP1-3. (JPG) [file pone.0030057.s001.jpg]

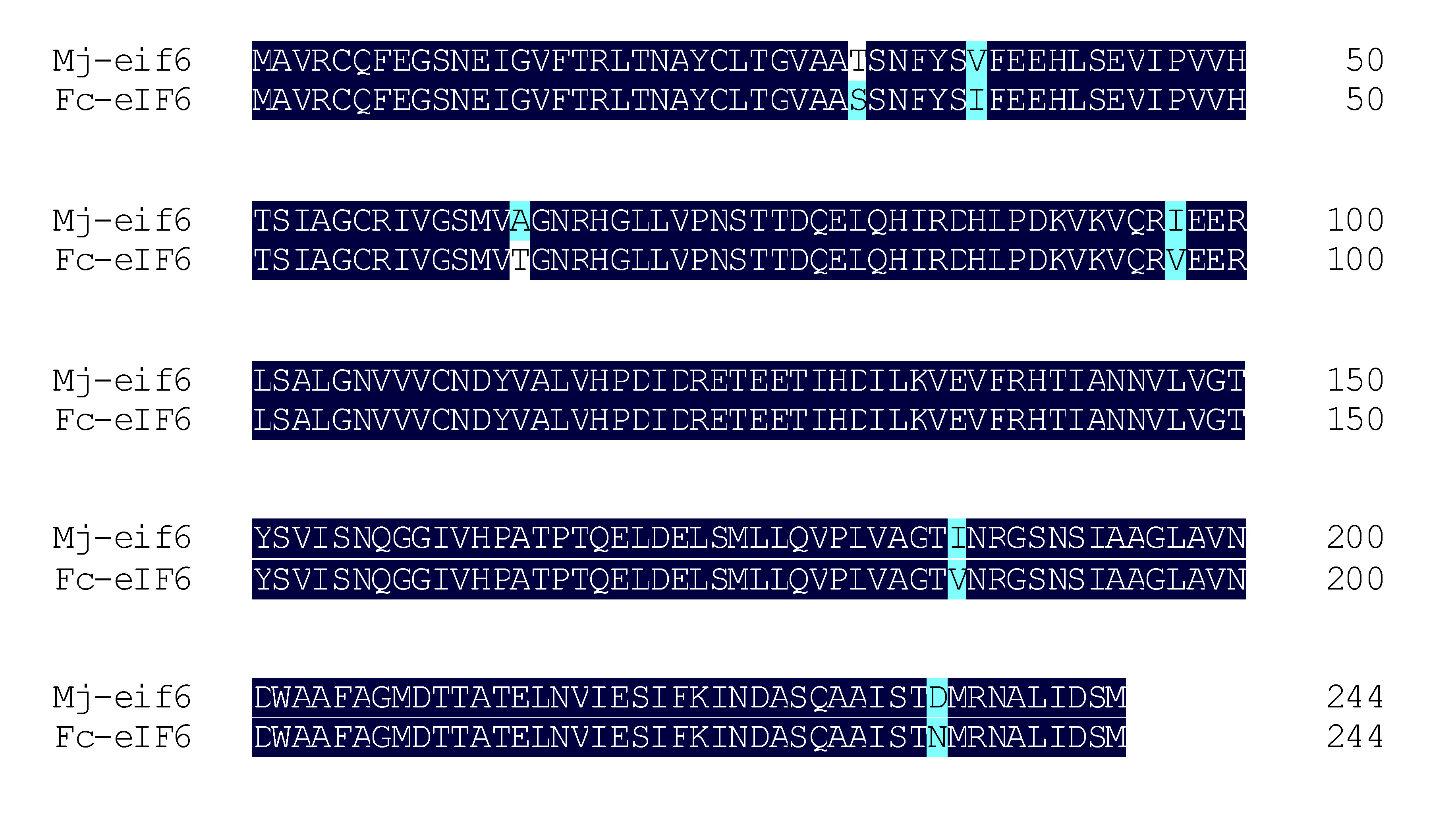

Supplement: Figure S2 — Multiple alignments of Fc-eIF6 (GenBank no. EU679001) with Mj- eIF6. (JPG) [file pone.0030057.s002.jpg]

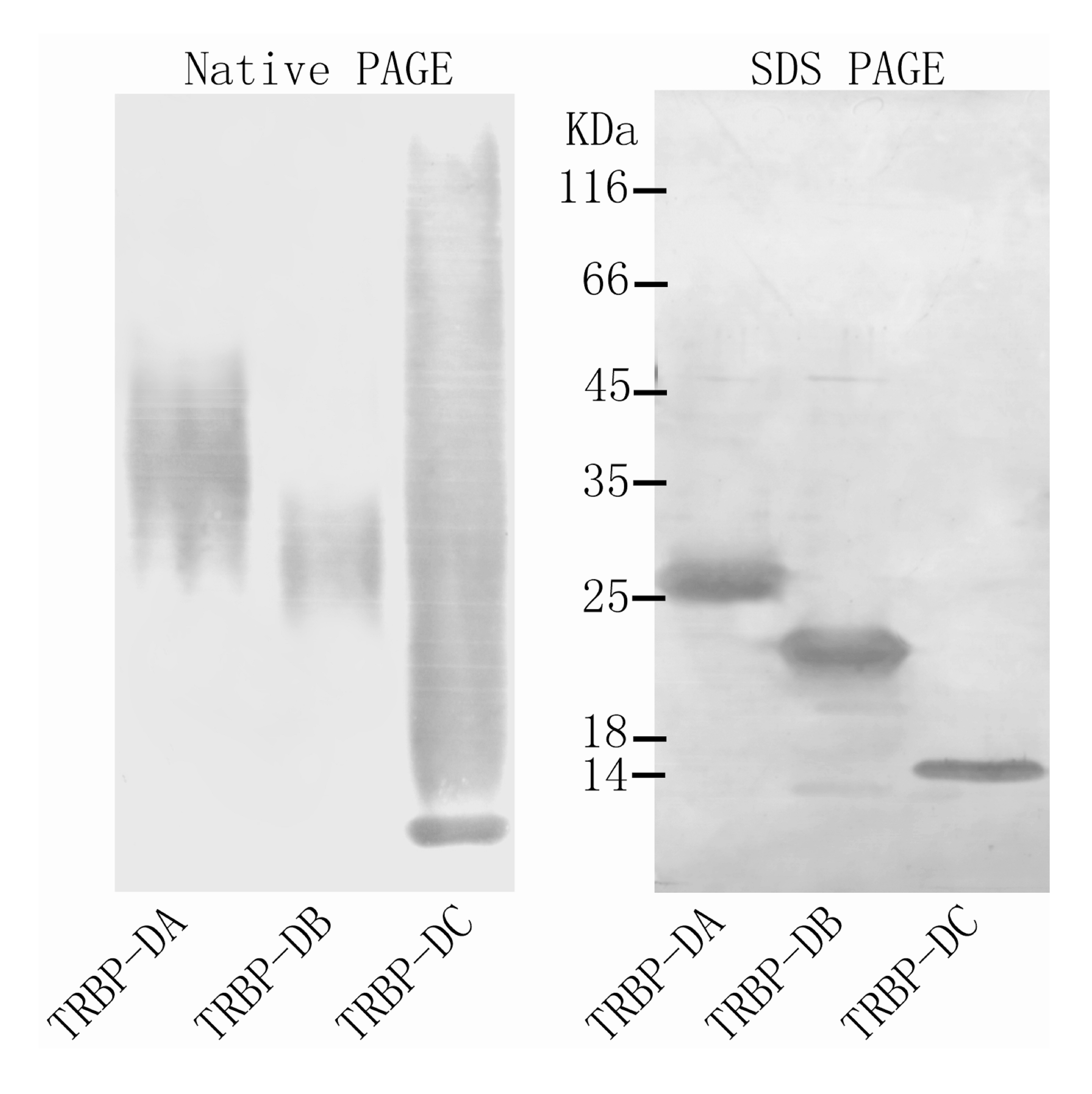

Supplement: Figure S3 — Native PAGE and Western blot was performed to confirm the dimerization of TRBP-DC(left panel). After Native PAGE of TRBP-DA,-DB and DC, the proteins in the PAGE gel were transferred onto a nitrocellulose membrane and were detected with TRBP antibody. TRBP-DA amd DB were used as control. TRBP-DC dimer exhibits a smear bands above the band of DC monomer. SDS-PAGE and Western blot were performed to verify the identity of the bands (right panel). (JPG) [file pone.0030057.s003.jpg]

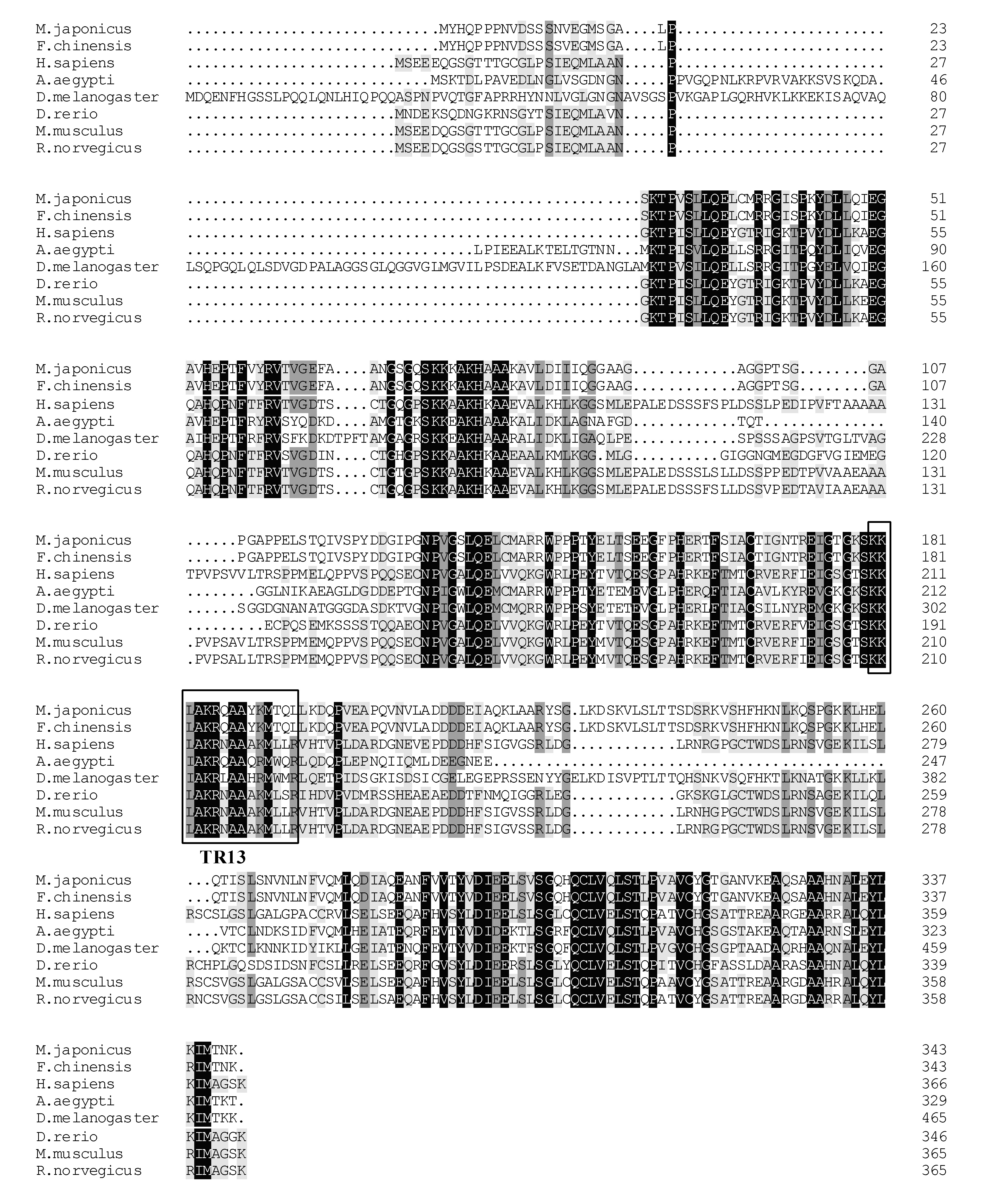

Supplement: Figure S4 — Multiple alignments of Mj-TRBP (GenBank no. HM149251) amino acid sequences from other animals. The TR13 sequence of human TRBP and the equivalent peptides in other animal are marked with a box. The following sequences were selected from GenBank: F. chinensis (EU679001), Aedes aegypti (XP_001659426.1), Danio rerio (NP_956291.1), Drosophila melanogaster (NP_609646.1), Homo sapiens (AAP36873.1), Mus musculus (AAH02028.1), Rattus norvegicus (NP_001030113.1), Xenopus tropicalis (NP_001025646.1). Dark shadow: identity = 100%; Grey shadow: identity≥75%; Light grey shadow: identity≥50%. The alignment was performed by DNAman 3.1. (JPG) [file pone.0030057.s004.jpg]
